# An ex vivo human osteochondral culture model

**DOI:** 10.1002/jor.24789

**Published:** 2020-07-10

**Authors:** Meike W. A. Kleuskens, Corrinus C. van Donkelaar, Linda M. Kock, Rob P. A. Janssen, Keita Ito

**Affiliations:** ^1^ Orthopaedic Biomechanics, Department of Biomedical Engineering Eindhoven University of Technology Eindhoven The Netherlands; ^2^ LifeTec Group BV Eindhoven The Netherlands; ^3^ Orthopaedic Center Máxima Máxima Medical Center Eindhoven/Veldhoven Eindhoven The Netherlands; ^4^ Value‐Based Health Care, Faculty of Paramedical Sciences Fontys University of Applied Sciences Eindhoven The Netherlands

**Keywords:** articular cartilage, ex vivo, explant, osteoarthritis, osteochondral

## Abstract

To reduce animal experimentation and to overcome translational issues in cartilage tissue engineering, there is a need to develop an ex vivo human tissue‐based approach. This study aims to demonstrate that a human osteochondral explant at different stages of osteoarthritis (OA) can be kept in long‐term culture while preserving its viability and composition. Osteochondral explants with either a smooth or fibrillated cartilage surface, representing different OA stages, were harvested from fresh human tibial plateaus. Explants were cultured for 2 or 4 weeks in a double‐chamber culture platform. The biochemical content of the cartilage of cultured explants did not significantly change over a period of 4 weeks and these findings were supported by histology. Chondrocytes mostly preserved their metabolic activity during culture and active bone and marrow were found in the periphery of the explants, while metabolic activity was decreased in the bone core in cultured explants compared to fresh explants. In fibrillated explants, chondrocyte viability decreased in the periphery of the sample in cultured groups compared to fresh explants (fresh, 94 ± 6%; cultured, 64% ± 17%, 2 weeks, and 69% ± 17%, 4 weeks; *P* < .05). Although biochemical and histological results did not show changes within the cartilage tissue, the viability of the explants should be carefully controlled for each specific use. This system provides an alternative to explore drug treatment and implant performance under more controlled experimental conditions than possible in vivo, in combination with clinically relevant human osteochondral tissue.

## 1 INTRODUCTION

Osteoarthritis (OA) is a degenerative condition of the joint, characterized by pain and reduced mobility caused by unfavorable changes in cartilage, subchondral bone, synovium, and other joint components.^1^ A complex interplay between the different components of the joint is responsible for this progressive disease.^2^ Researchers have studied OA in various culture models, such as cell monolayers, cell cocultures, 3D cell constructs, and ex vivo explants.^3^, ^4^, ^5^, ^6^ Each model has its own advantages and drawbacks, but since it has been generally accepted that more joint structures than only cartilage play a role in OA, there is an increasing interest in the use of osteochondral culture models.^7^ The use of osteochondral explants has several advantages over cartilage‐only explants. First, cartilage is less damaged, because it is not detached from the bone and this ensures a natural mechanical constraint against adverse deformation. Second, it has been established that bone can influence the biological behavior of cartilage. Data from previous research shows that collagen type II gene expression was significantly higher in cultured cartilage from osteochondral explants than from cartilage‐only models.^7^ Also, it was shown that subchondral bone was the main source of secreted factors for chondrogenesis by human bone marrow mesenchymal stem cells.^8^ In summary, it seems that the attachment to the bone is beneficial for cartilage, making it a more representative culture system.

Recently, a separated two‐chamber culture model was developed in which the cartilage of a healthy porcine osteochondral plug could be kept viable and biochemically and biomechanically intact for at least 56 days in culture.^9^ The model mimics the native condition more than previous studies, by separating the nutrient supply of cartilage and bone by two separate media compartments containing tissue‐specific medium. It was shown that this positively influenced cartilage health and viability.^9^ In this platform, diffusion of signaling molecules between the tissues could only occur through the subchondral bone plate at the bone‐cartilage interface, as there is no external communication between the medium compartments. This ex vivo culture method might be a promising new way of assessing osteochondral and cartilage repair strategies.

The aforementioned study used osteochondral explants from 6 to 8 months old pigs of which the cartilage has a healthy and smooth appearance.^9^ However, the patients most likely to receive treatment will be middle‐aged and their cartilage will not be healthy anymore with behavior dissimilar to that of young healthy porcine cartilage. The extent of this difference and the consequences thereof for the interpretation of experimental studies is unknown. Translation of intervention studies to clinical practice may benefit from ex vivo studies using human osteochondral explants. The first step towards such evaluations is to explore what the effect of ex vivo culture is on (early) OA osteochondral explants. Degenerated OA cartilage has a different structure than healthy cartilage. Degenerated cartilage is softer, and the surface becomes fibrillated or fissured. The tidemark deteriorates and irregularities might be observed.^10^ In the early stages of OA, chondrocyte proliferation, and proteoglycan (PG) synthesis are increased. The cartilage surface suffers from a loss of PGs and cleavage of collagen type II, leading to an increase in water content. At later stages, the breakdown of matrix components exceeds the synthesis rates.^11^ It has been observed that healthy cartilage explants and normal looking cartilage explants from a knee with focal degeneration signs had similar Mankin scores, as well as similar DNA content, sulfated glycosaminoglycan (sGAG) content, sulfate incorporation rate and release of sGAGs and newly synthesized sGAGs after 4 days of culture.^12^ Therefore, leftover patient tissue can be used to study different stages of degeneration. In order to assess the effect of clinical interventions using such culture systems, it is important that the explant tissue is stable in terms of biochemical content, distribution, and viability in longer‐term cultures. Previous studies culturing osteochondral explants harvested from total knee replacement leftover tissue have shown its potential as a treatment evaluation system, but did not closely look into cell viability,^13^, ^14^ nor biochemical analysis,^13^ or showed decreased sGAG content when explants were cultured in one medium compartment.^14^

Therefore, the aim of this study was to demonstrate that osteoarthritic human osteochondral explants can be maintained in long‐term culture in terms of viability and composition. To do so, human osteochondral explants at different stages of OA were cultured in a system with separated cartilage and bone compartments for a period up to 4 weeks, followed by analysis of biochemical content, histology, and viability.

## 2 MATERIALS AND METHODS

### 2.1 Explants

Human tibia plateaus were collected from patients undergoing total knee replacement at Màxima Medical Centre (Eindhoven, the Netherlands), approved by the Local Medical Ethical Committee (METC, number N16.148). Of the least affected compartment of the plateaus, tissue was isolated from two locations, that is, with a smooth, healthy‐looking layer of cartilage (smooth surface [S]) and with a fibrillated, degenerate looking layer of cartilage (fibrillated surface [F]), representing a lower and higher grade of OA, respectively. To confirm the ability of macroscopically assigning explants to these two groups, fresh explants of both groups (S, n = 6 and F, n = 9, of 7 subjects, 4 female and 3 male, aged 72 ± 6 years [mean ± SD], range 63‐84 years) were prepared for histological assessment. To assess osteochondral explant stability in culture, explants from 10 other subjects were used (six females and four males, aged 67 ± 9 years (mean ± SD), range 54‐82 years). For each fresh explant, another explant of the same subject was cultured for either 2 or 4 weeks, except for one smooth and two fibrillated fresh explants, for which two explants of the same subject were cultured (one for 2 weeks and one for 4 weeks). Fresh explants (W0, S, n = 8 and F, n = 9) were isolated and immediately processed. Remaining explants were cultured for 2 weeks (W2, S, n = 4 and F, n = 6) or 4 weeks (W4, S, n = 5 and F, n = 5) as described below, and thereafter prepared for further analysis.

### 2.2 Methods: initial macroscopic classification

#### 2.2.1 Sample preparation

Osteochondral explants, 10 mm in diameter, were isolated using a custom‐made trephine drill while cooling with 4°C phosphate‐buffered saline (PBS; Sigma‐Aldrich, Zwijndrecht, the Netherlands) supplemented with 1% penicillin/streptomycin. The explant bone length was cut to 4 mm and the cartilage layer of the explants was covered with a 3% agarose (Sigma‐Aldrich) solution to prevent glycosaminoglycan (GAG) leakage. Explants were fixed in 3.7% formalin (Merck KGaA, Darmstadt, Germany) for 24 to 48 hours and then decalcified in a 12.5% EDTA solution (Sigma‐Aldrich) at 37°C for 2 to 4 weeks. EDTA solution was refreshed twice a week. The explants were then fixed again for 24 hours, cut in half, dehydrated, and embedded in paraffin using a tissue processor (Microm, Walldorf, Germany). Sections of 10 µm thickness were prepared for each explant using a microtome (Leica, Wetzlar, Germany) and stored at room temperature until use.

#### 2.2.2 Safranin‐O/fast green staining

Sections of the osteochondral explants were stained for GAG and collagen with Safranin‐O/fast green. In short, sections were rehydrated, washed, and stained in Weigert's Iron Hematoxylin (Sigma‐Aldrich) for 8 minutes, 0.01% fast green solution (Sigma‐Aldrich) for 2 minutes and 0.1% Safranin‐O solution (Sigma‐Aldrich) for 8 minutes. Sections were briefly rinsed in 95% alcohol and dehydrated in three changes of 100% alcohol and two changes of xylene (Merck). Sections were mounted using Entellan (Merck) and visualized using brightfield microscopy (Observer Z1, Carl Zeiss, Jena, Germany).

#### 2.2.3 Picrosirius red staining

Sections of the osteochondral explants were stained for collagen using picrosirius red. In short, sections were rehydrated, washed, and stained in a 0.1% picrosirius red solution (Sigma‐Aldrich) for 1 hour, rinsed in 0.1%, and 0.5% glacial acetic acid solutions (Merck) for 1 minute each, and dehydrated to xylene. Sections were mounted using Entellan and visualized using brightfield microscopy (Observer Z1).

#### 2.2.4 Data analysis

The histology sections were used to score the OA grade using the Mankin scoring system.^15^, ^16^ This was done independently by three scorers on anonymized and randomized pictures, and the final score per explant was averaged. Data are presented as individual scores per OC explant with a horizontal line representing the mean. Statistical analysis was performed with Prism 5 (GraphPad Software, La Jolla, CA, www.graphpad.com). A nonparametric Mann‐Whitney *U* test was conducted to compare Mankin scores of smooth and fibrillated cartilage explants. A nonparametric test was chosen because of the small sample size. Statistical significance was assumed when *P* < .05.

### 2.3 Methods: stability of the explants during culture

#### 2.3.1 Explant harvest and culture

**Figure 1:**
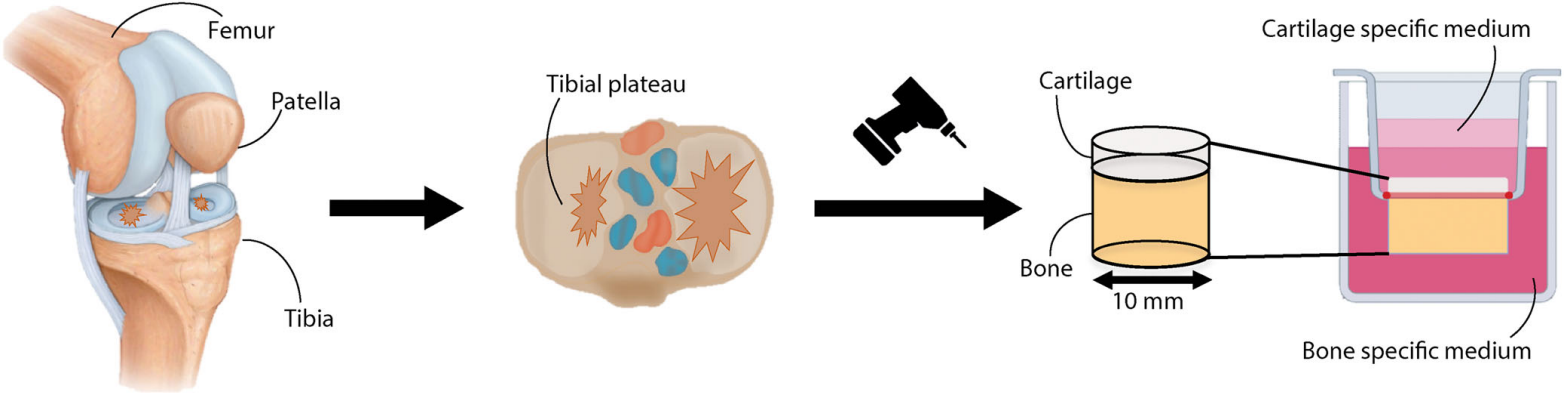
Osteochondral explant harvesting and culture system [Color figure can be viewed at wileyonlinelibrary.com]

Osteochondral explants were prepared as described for the fresh specimens, immediately placed in inserts, and washed with PBS until further use (Figure 1). The inserts are part of the culture platform used, which provided separate medium compartments for the bone and cartilage tissue (LifeTec Group BV, Eindhoven, the Netherlands).^9^ Explants were then cultured in separate wells provided with 3 mL bone medium, consisting of DMEM high glucose (14966; Gibco, Bleiswijk, the Netherlands) supplemented with 10% fetal bovine serum (FBS; Gibco), 1% penicillin/streptomycin (Lonza, Basel, Switzerland), 25 μg/ml fungin (InvivoGen, Toulouse, France), 50 μg/mL l‐ascorbic acid‐2‐phosphate (Sigma‐Aldrich) and 10 mM β‐glycerophosphate (Sigma‐Aldrich) in the bone compartment and 2.5 mL cartilage medium, consisting of DMEM high glucose (14966) supplemented with 1% penicillin/streptomycin, 25 μg/mL fungin, 40 μg/mL l‐proline (Sigma‐Aldrich), 50 μg/mL l‐ascorbic acid‐2‐phosphate, and 1% ITS + Premix (Corning, Fisher Scientific, Landsmeer, NL) in the cartilage compartment. The medium was refreshed every 3 to 4 days and explants were cultured at 37°C and 5% CO_2._

#### 2.3.2 Biochemistry

A 1/8 wedge of each fresh and cultured explant was used for biochemical analysis. The cartilage was separated from the bone with a scalpel and stored at −80°C until use. Cartilage samples were blotted dry, weighed (wet weight), lyophilized, and weighed again to determine their dry weight. Water content was calculated by dividing the weight loss during lyophilization by the wet weight. Then samples were digested overnight at 60°C in a papain digestion buffer (100 mM phosphate buffer, 5 mM L‐cysteine, 5 mM ethylene diamine tetra‐acetic acid (EDTA) and 140 µg/mL papain, all from Sigma‐Aldrich). sGAG content was determined using a dimethyl methylene blue (DMMB) assay with shark cartilage chondroitin sulfate as a reference. The hydroxyproline (HYP) content, as measure for collagen content, was determined with a chloramine‐T assay with trans‐4 hydroxyproline (Sigma‐Aldrich) reference. DNA content was determined from the digested samples using the Qubit dsDNA high sensitivity kit (Invitrogen, Waltham, MA) according to the manufacturer's instructions.

#### 2.3.3 Frozen sections‐preparation

Cartilage of a 1/4 wedge of each explant was separated from the bone using a scalpel. The cartilage piece was embedded in Tissue‐Tek O.C.T. compound (Sakura, Alphen aan den Rijn, the Netherlands) and frozen on dry ice. Sections of 10 µm thickness were prepared for each explant using a cryostat cryotome (Fisher Scientific, Landsmeer, the Netherlands) and stored at −20°C until further use.

#### 2.3.4 Safranin‐O/fast green or Picrosirius red staining

After fixation in 3.7% formalin for 5 minutes, frozen sections of the cartilage were prepared and imaged in a similar fashion as the fresh OC explant sections.

#### 2.3.5 Viability

A pie‐shaped quarter of each explant was used immediately after isolation or at end of culture for 3‐(4,5‐dimethylthiazolyl‐2)‐2,5‐diphenyltetrazoliumbromide (MTT; Invitrogen Molecular Probes, Eugene, OR) analysis, in which the yellow tetrazole is reduced to purple formazan in metabolically active cells. A 5 mg/mL solution of MTT in PBS was diluted in DMEM to reach a final concentration of 0.37 mg/mL. samples were washed in PBS and incubated in 1 mL of MTT solution for 90 minutes at 37°C. Images were acquired using a Keyence microscope (VHX‐500FE, Osaka, Japan). Images of the bone and cartilage were compared between time points.

Additionally, cryosections were stained for lactate dehydrogenase (LDH) activity according to a protocol adopted from Stoddart et al.^17^ An LDH solution was freshly prepared on the day of staining, by adding 60 mM lactic acid (Sigma‐Aldrich) and 1.75 mg/mL β‐nicotinamide adenine dinucleotide (Sigma‐Aldrich) to a polypep solution consisting of 40% polypep (Sigma‐Aldrich) and 50 mM gly‐gly (Sigma‐Aldrich) in H_2_O. The pH was adjusted to 8.0 by adding 5 M sodium hydroxide (NaOH). Just before use, 3 mg/mL nitro blue tetrazolium (NBT; Sigma‐Aldrich) was added. Thawed cryosections were incubated in the LDH solution for 4 hours at 37°C, in a moisturized chamber and protected from light. Afterward, sections were rinsed with water (50°C), PBS and fixed with 70% ethanol (EtOH) for 5 minutes. Then the sections were stained with a 1% propidium iodide (PI; Invitrogen Molecular Probes) in 0.1% PBS‐triton solution for 5 minutes. Samples were rinsed three times in PBS and mounted with Mowiol (Sigma‐Aldrich). Samples were visualized using brightfield microscopy for LDH and fluorescent microscopy for PI (DMi8; Leica). Living and dead cells were counted using ImageJ software, by counting the number of cells in the brightfield channel (LDH/alive), the fluorescent channel (PI) and the colocalizations, and then subtracting the number of colocalizations from the number of cells in the fluorescent channel, to obtain the number of dead cells. The fraction of dead cells was calculated by dividing the number of dead cells by the total number of cells.

#### 2.3.6 Data analysis

Biochemical assay and LDH assay data are presented as scatterplots with a horizontal line representing the mean. Statistical analysis was performed with Prism 5. A Kruskal‐Wallis test was conducted to examine the effect of culture length, and if appropriate, a Dunn's posthoc test was done to discriminate between groups. A nonparametric test was chosen because of the small sample size. Statistical significance was assumed when *P* < .05.

## 3 RESULTS

### 3.1 Initial macroscopic classification

**Figure 2:**
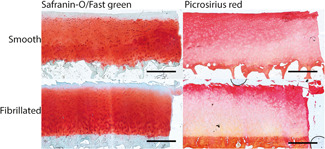
Representative examples of sections stained with Safranin‐O/fast green (left) and picrosirius red (right) for smooth and fibrillated explants immediately after isolation. Scalebar = 1 mm [Color figure can be viewed at wileyonlinelibrary.com]

**Figure 3:**
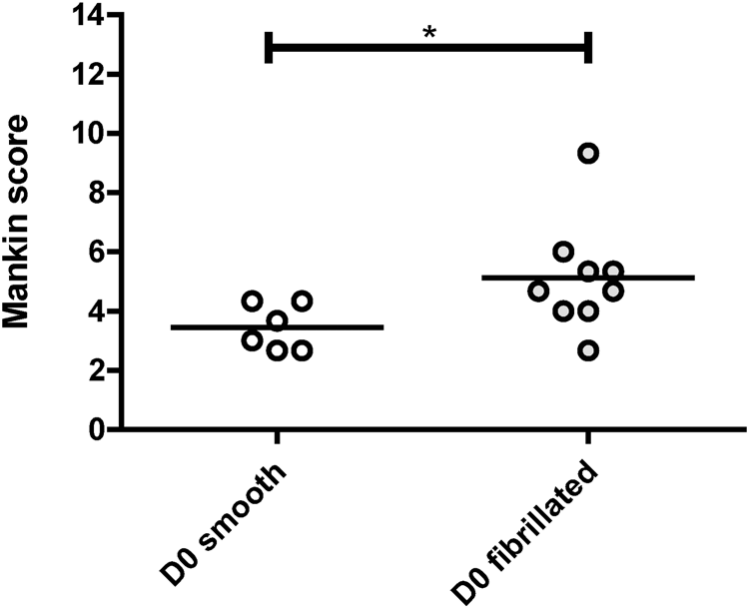
Mankin score for fresh smooth explants was significantly lower than for fibrillated explants (*P* < .05)

The main differences observed between smooth and fibrillated explants were surface smoothness and cellular density. Representative images of the Safranin‐O/fast green and picrosirius red‐stained sections are shown in Figure 2. The average Mankin score for smooth surface cartilage was significantly lower than for fibrillated cartilage at the moment of harvesting (D0) (S, 3.4 ± 0.8 and F, 5.1 ± 1.9; Figure 3).

### 3.2 Stability of the explants during culture

**Figure 4:**
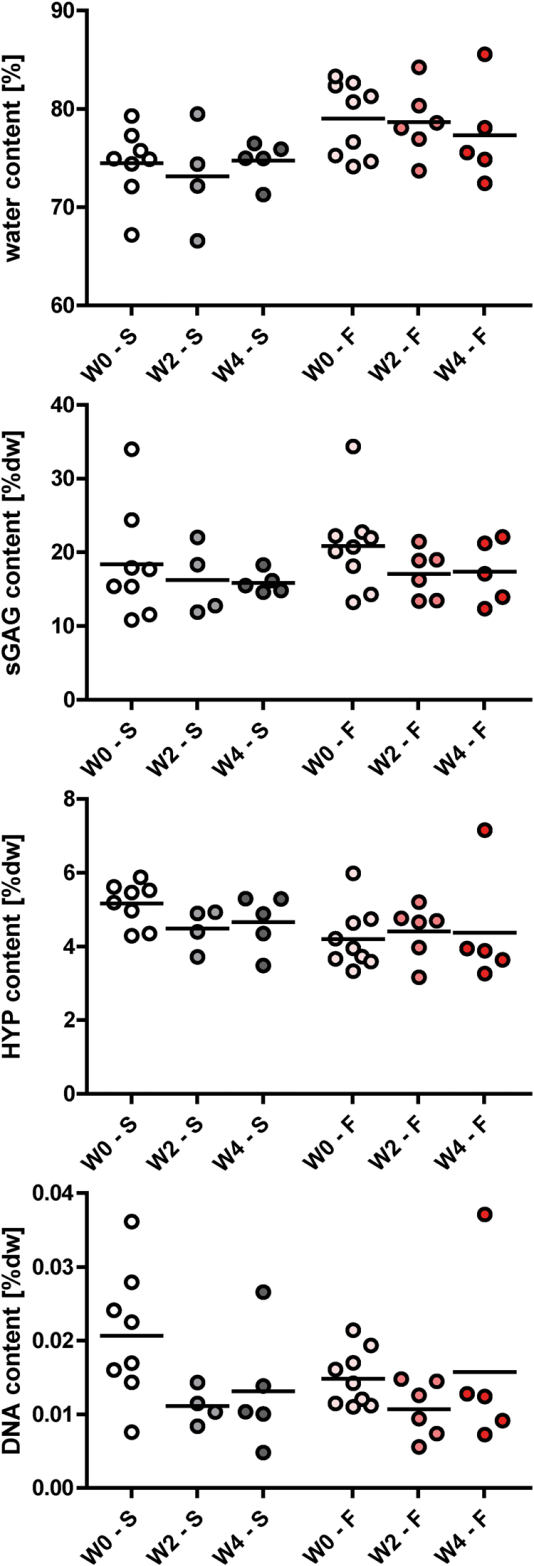
Water, sGAG, HYP, and DNA content of the cartilage. F, fibrillated cartilage surface; HYP, hydroxyproline; S, smooth cartilage surface; sGAG, sulfatedglycosaminoglycan; W0, fresh; W2, 2 weeks of culture; W4, 4 weeks of culture [Color figure can be viewed at wileyonlinelibrary.com]

Culturing both smooth and fibrillated osteochondral explants did not lead to significant changes in water, sGAG, HYP and DNA content of the cartilage after 2 and 4 weeks (W2, W4) compared to W0, for both smooth (‐S) and fibrillated (‐F) explants (Figure 4), with only a nonsignificant decreasing trend over time in DNA content in the smooth cartilage.

**Figure 5:**
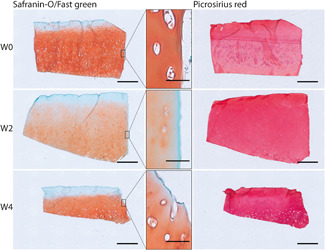
Representative examples of sections stained with Safranin‐O/fast green (left) and picrosirius red (right) for smooth explants of W0, W2, and W4 groups. W0, fresh; W2, 2 weeks of culture; W4, 4 weeks of culture; left side of each image is the core of the explant, right side is the periphery of the explant. Highlighted boxes show zoomed in area of the periphery of the explants. Scalebar = 1 mm for the overview images and scalebar = 0.1 mm for the highlighted boxes [Color figure can be viewed at wileyonlinelibrary.com]

**Figure 6:**
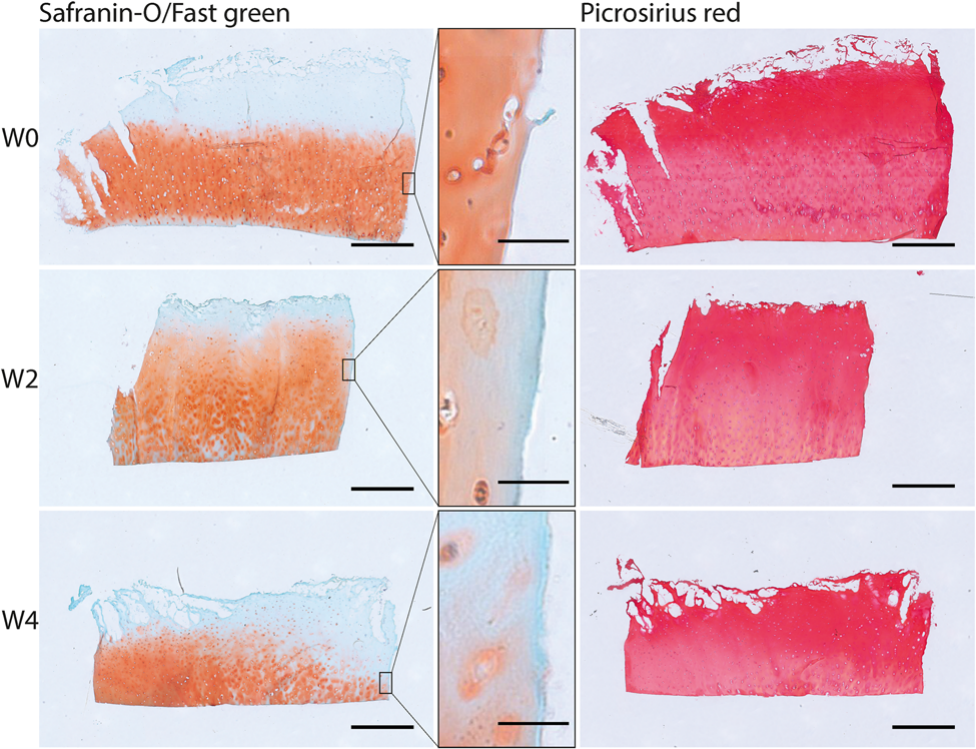
Representative examples of sections stained with Safranin‐O/fast green (left) and picrosirius red (right) for fibrillated explants of W0, W2, and W4 groups. W0, fresh; W2, 2 weeks of culture; W4, 4 weeks of culture; Left side of each image is the core of the explant, right side is the periphery of the explant. Highlighted boxes show zoomed in area of the periphery of the explants. Scalebar = 1 mm for the overview images and scalebar = 0.1 mm for the highlighted boxes [Color figure can be viewed at wileyonlinelibrary.com]

Safranin‐o/fast green and picrosirius red images confirmed the biochemical stability of the cartilage tissue in culture. Over time, no visible changes in GAG and collagen distribution were observed within the groups, apart from some GAG loss at the periphery of mainly the fibrillated explants (Figures 5 and 6).

**Figure 7:**
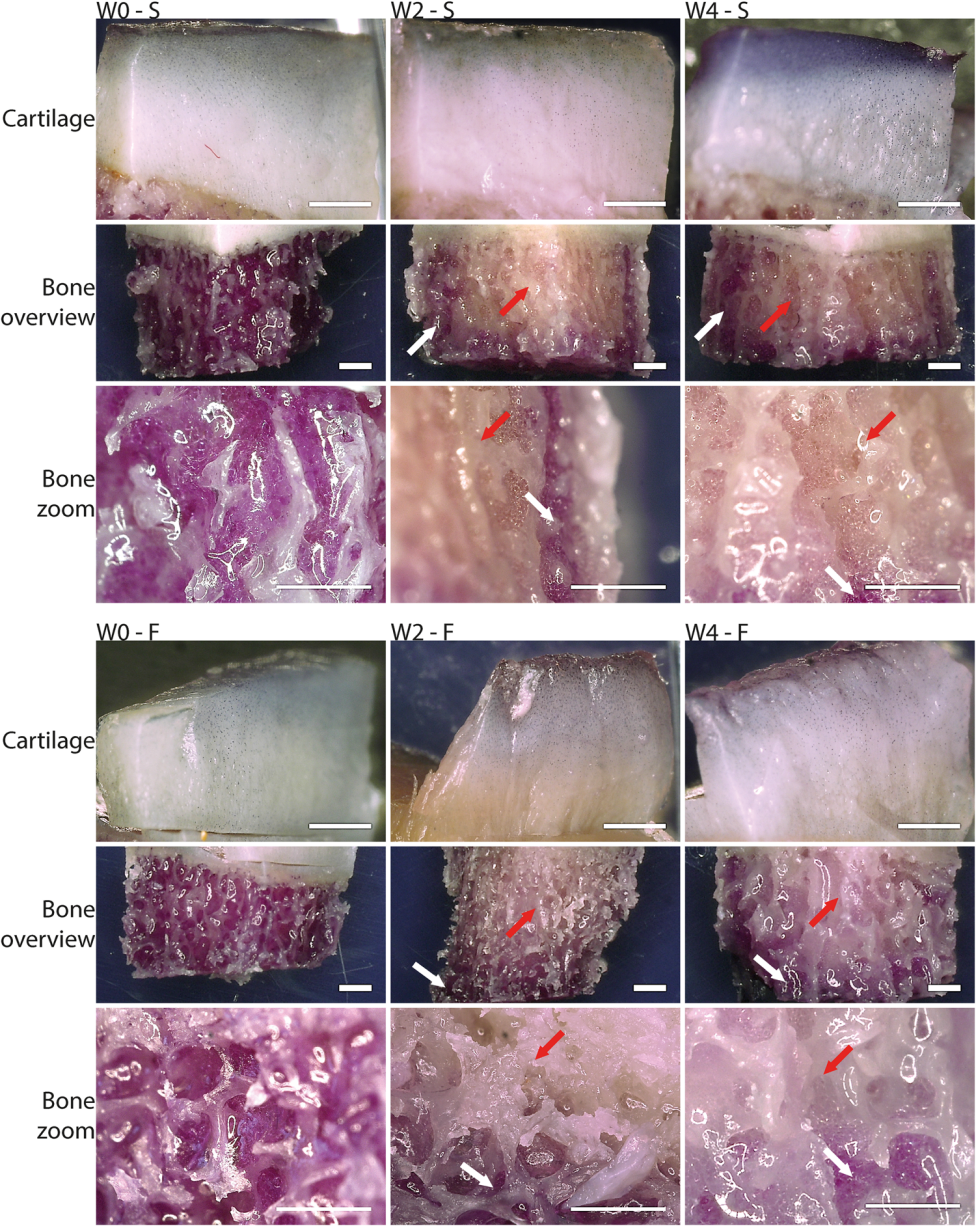
Representative images of MTT analysis for metabolic activity within smooth and fibrillated explants before and after culture. White arrows help to show bone regions that are metabolically active (purple bone area). Red arrows help to show bone regions that are less metabolically active (yellow bone area). F, fibrillated cartilage surface; S, smooth cartilage surface; W0, fresh; W2, 2 weeks of culture; W4, 4 weeks of culture. Scalebar = 1 mm [Color figure can be viewed at wileyonlinelibrary.com]

**Figure 8:**
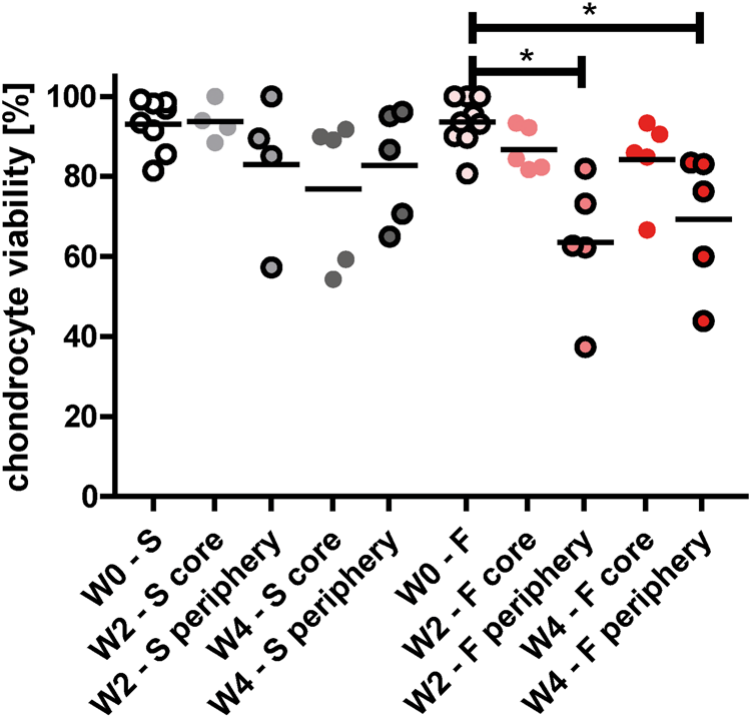
Chondrocyte viability within the explants (* = *p* < .05) [Color figure can be viewed at wileyonlinelibrary.com]

Cartilage and bone tissue were viable immediately after isolation (W0). While the entire cartilage and the bone tissue at the periphery of the explants remained viable during culture, a decreased viability was observed in the bone core of the explants (Figure 7). This effect was already observed at W2, and was similar for W4 in both smooth and fibrillated explants. The bone death in the core of the explants did not influence the chondrocyte viability in the core, as MTT staining of cartilage shows similar results for W0, W2, and W4. A closer look at the chondrocyte viability in a LDH/PI assay indeed revealed no loss of viability in both core and periphery of smooth explants and the core or fibrillated explants over time (Figure 8). In the periphery of fibrillated explants, however, chondrocyte viability was decreased after 2 and 4 weeks (W2, 64% ± 17% and W4, 69% ± 17% compared to W0, 94% ± 6%; *P* < .05). Note the distribution within the groups, indicating that cartilage of some explants had much lower viability than others.

## 4 DISCUSSION

This study demonstrated a culture system that could be used to maintain human osteochondral explants with different OA grades for at least 28 days. Biochemical fractions and histological appearance remained constant (Figures 4‐6). Although chondrocyte viability was stable in smooth cartilage explants, in the fibrillated samples decreased viability in the periphery was observed (Figure 8). In the bone and marrow tissue, the central core of the explant did not survive (Figure 7). However, this is less consequential as cartilage treatments will be applied to the core of the explant and the bone core will be replaced by bone treatment or may be resolved by other means (see below).

In this study, a two‐compartment culture platform was used to culture bone and cartilage, each in their own specific medium, where crosstalk is only possible through the bone—cartilage interface. A one‐compartment study conducted by de Vries‐van Melle et al.^18^ showed that the presence of subchondral bone resulted in a more representative gene expression profile of cartilage‐related genes compared to a cartilage‐only explant. However, the expression of cartilage‐related genes still decreased over a 28‐day culture period.^18^ Furthermore, a human osteochondral explant culture study by Yeung et al. showed a large decrease in cartilage sGAG content in 4 weeks, indicating their culture conditions, Ø3 mm explants in one medium type without separation of cartilage and bone, were suboptimal for sGAG retention in the tissue.^14^ Also, previous co‐culture models of bone and cartilage have shown that factors secreted into the culture medium by sclerotic osteoblasts and mononucleated cells present in the bone marrow, influence chondrocyte metabolism as indicated by a reduction of cartilage matrix synthesis.^19^, ^20^ Schwab et al.^9^ hypothesized that such issues might be solved by mimicking the in vivo situation using a two‐compartment culture system in which the crosstalk between bone and cartilage is restricted to their interface, and indeed they showed improved sGAG retention in a two‐compartment setup where bone and cartilage were each supplied with their own specific medium, compared to a one‐compartment setup. Thereafter, other studies using the same two‐compartment culture system have confirmed that sGAG content was retained over 56 days of culture in porcine explants, but that it decreased in equine explants over 29 days. Thus, demonstrating that application of this culture platform approach to other species requires validation.^9^, ^21^

The biochemical fractions measured in the current study are in agreement with other studies and importantly, there were no changes in biochemical composition over time for both smooth and fibrillated cartilage (Figure 4).^22^, ^23^ No significant difference in sGAG and HYP content was detected between smooth and fibrillated cartilage, although it was observed that initially, fibrillated cartilage had on average a (nonsignificant) slightly higher water and relative sGAG content and a lower relative HYP content compared to the smooth cartilage. This is in agreement with the commonly seen changes in early OA, which include roughening of the superficial cartilage layer, lowering the collagen content and therefore HYP content (hence classification as “fibrillated” in this study), and an increase in PG synthesis (hence larger relative sGAG content).^11^ Histological analyses showed some minor GAG loss at the edges of (mainly fibrillated) explants, which was not seen in biochemical analysis (Figures 5, 6). This effect is probably the result of drilling the explants, and not due to the culturing protocols, because it is already apparent in the first measurement point and the effect does not worsen over time. Besides, the effect was not seen in the biochemical analysis indicating that this GAG loss was the only minor.

Similar to the porcine explants that were used in previous studies, bone viability in the human explants decreased during culture.^9^ The MTT assay shows that viable cells are still present in the periphery of the explant, while in the central bone core viability decreases (Figure 7). This is likely not only caused by the lack of vital function of the blood cells and platelets, but also by insufficient nutrition supply. The bone of the elderly patients contained fatty marrow, which may hamper diffusion. The interrupted blood and marrow circulation within the bone may have prevented the nutrients from flowing through the trabecular bone. Despite the cell death in the core of the bone, chondrocyte viability in the core was maintained, suggesting there was no adverse influence on the cells in the cartilage tissue. It was observed however that chondrocyte viability in the periphery of the fibrillated explants dropped to just over 60% in two out of five samples after 2 weeks, after which it stayed constant during the remainder of the culture period (Figure 8). Excessive shear strain or temperature rise during drilling the explant may have been responsible for this effect. Shear is expected to be higher in fibrillated than in healthy cartilage, as even mildly fibrillated cartilage loses transverse stiffness and may become more susceptible to deformation during drilling.^24^ Before culturing fibrillated cartilage in a study that demands high viability throughout the sample, this requires attention and may be solved by coring instead of drilling.^25^ Attempts to clear the tissue from the fatty marrow by washing or cleaning with PBS or saline solution directly after isolation were not successful. In future experiments, osteochondral implants might be tested in this culture system. The central core of the bone and cartilage will be removed when osteochondral implants will be inserted, eliminating the bone viability issues. Alternatively, if only cartilage treatments are to be tested, the central core of bone may also be removed from below.

Using human instead of animal tissue to study osteoarthritis has some major advantages. First, by using human tissue there is no need to discuss or compensate for differences in size, anatomy, biomechanics, and cartilage thickness. Second, using the older, human tissue there is no need to compensate for the structural and compositional differences between cartilage of young and older individuals, either animal or human. Older cartilage tissue may contain less water, be more fibrillated, more crosslinked, stiffer, and with a different GAG/collagen ratio.^26^ Moreover, cellular activity decreases with age, which might lead to a different treatment response.^26^ Third, using human patient tissue eliminates the need for inducing OA in a mechanical or chemical fashion, providing a more representative and natural disease model. On the other hand, there is variability between and within tissue of the patients. Age, sex, genetics, and environmental factors might all play a role in OA. The tissue that is isolated in the operating theatre is of varying quality leading to material which is much more variable in its composition and behavior than animal material. Moreover, biochemical content of cartilage is quite variable across the joint as exemplified by the large standard deviations in contents in D0 samples for both the smooth and fibrillated groups (Figure 4). It was previously reported that sGAG content of the cartilage within a joint may vary up to 50%.^27^, ^28^ A possible way to overcome these effects is to use smaller size explants, such as for example Yeung et al. who cultured human osteochondral explants of Ø3 mm with a Ø1 mm defect.^14^ This would allow for a larger number of explants per donor; however, this explant and defect size will limit the possibilities for analysis techniques, and defect size is far from clinically relevant. The system presented here with Ø10 mm osteochondral explants allows for experiments in which the core may be removed and replaced by an implant to explore integration with the environment, while at the same time it provides a method for culturing the osteochondral explants while maintaining their sGAG content. In summary, this human ex vivo culture system is an addition to the existing setups, providing a true disease model, though results must be interpreted with care.

In this study, human tibia plateaus were used as source for the osteochondral explants. Although the femoral condyles are more susceptible to focal defects,^29^, ^30^, ^31^ the quality of the cartilage in tibia plateaus is better on average, and plateaus contain a thicker bone layer and larger relatively flat areas. Smooth surface explants were harvested from the outer rim of the tibia plateau, covered by the meniscus, while fibrillated surface explants were harvested from the center of the lateral tibia plateau, where cartilage is in direct contact with femoral cartilage and pressures are higher.^32^ Explants that were assigned to the smooth explants group had a Mankin score of 3.4 ± 0.8 (with 0/6 assigned a Mankin score >5.1) and fibrillated explants had a Mankin score of 5.1 ± 1.9 (1/8 assigned a Mankin score <3.4), indicating that this assignment based on visible inspection was appropriate (Figures 2 and 3). Previous research has shown that cartilage explants from these areas are different in their PG and HYP content in a similar fashion as found in the current study.^27^, ^28^, ^32^ As in this study the aim was to explore the stability of both smooth and fibrillated cartilage explants, and not to compare between groups, these differences do not affect the conclusions. However, this confounding factor needs to be considered in forthcoming studies where responses between tissues with various degrees of OA are to be compared. Another consequence of the large spatial variation is that pairwise comparison of biochemical content measured in a D0 sample with the biochemical content in a cultured sample, even if they come from adjacent locations in the same joint, is inappropriate.^27^, ^28^ Therefore, samples were randomly distributed between groups of different culture duration, and results were statistically analyzed using tests that assume independent samples, and not paired samples. It seems that for some donors, chondrocyte viability tends to decrease over time in both core and periphery of the smooth explants. A division is observed between explants with ~90% viability after 4 weeks and explants in which viability decreases to under 70% (in both core and periphery, Figure 8), indicating that in further research, close attention has to be paid to cell viability, and to selection of explants for further evaluation accordingly.

This study demonstrates that human osteochondral explants with a smooth cartilage layer can be kept in culture for at least 4 weeks while preserving cartilage viability and composition. For fibrillated explants, more care is needed and specifically viability of the periphery of the cartilage needs to be carefully monitored for each specific use. This ex vivo platform can be developed into a more complete joint model by for example adding more joint components such as synovium or synovial fluid, and by adding mechanical stimulation. Existing and novel treatments can be tested and evaluated, by for example adding components or drugs to the culture medium. To evaluate implant performance, both osteochondral and chondral defects can be created in the explants, and implants can be inserted to evaluate integration and regeneration capacities.^1^

## AUTHOR CONTRIBUTIONS

MWAK designed and conducted the experiments, interpreted and analyzed the data, and wrote the manuscript. CCvD and KI secured the funding, contributed to experiment design, data interpretation and manuscript review. RPAJ contributed to tissue procurement and harvesting as well as data interpretation and manuscript review. LMK provided expertise with the bioreactors and contributed to data interpretation and manuscript review. All authors have read and approved the final submitted manuscript.
